# Repeated, Intermittent Social Defeat across the Entire Juvenile Period Resulted in Behavioral, Physiological, Hormonal, Immunological, and Neurochemical Alterations in Young Adult Male Golden Hamsters

**DOI:** 10.3389/fnbeh.2016.00110

**Published:** 2016-06-10

**Authors:** Wei-Chun Yu, Ching-Yi Liu, Wen-Sung Lai

**Affiliations:** ^1^Department of Psychology, National Taiwan UniversityTaipei, Taiwan; ^2^Graduate Institute of Brain and Mind Sciences, National Taiwan UniversityTaipei, Taiwan; ^3^Neurobiology and Cognitive Science Center, National Taiwan UniversityTaipei, Taiwan

**Keywords:** repeated intermittent social defeat, social threat, juvenile, male golden hamsters, cortisol, pro-inflammatory cytokines, monoamine neurotransmitters, adolescent bullying

## Abstract

The developing brain is vulnerable to social defeat during the juvenile period. As complements of human studies, animal models of social defeat provide a straightforward approach to investigating the functional and neurobiological consequences of social defeats. Taking advantage of agonist behavior and social defeat in male golden hamster, a set of 6 experiments was conducted to investigate the consequences at multiple levels in young adulthood resulting from repeated, intermittent social defeats or “social threats” across the entire juvenile period. Male hamsters at postnatal day 28 (P28) were randomly assigned to either the social defeat, “social threat”, or arena control group, and they correspondingly received a series of nine social interaction trials (i.e., either social defeat, “social threat”, or arena control conditions) from P33 to P66. At the behavioral level (Experiment 1), we found that repeated social defeats (but not “social threats”) significantly impacted locomotor activity in the familiar context and social interaction in the familiar/unfamiliar social contexts. At the physiological and hormonal levels (Experiments 2 and 3), repeated social defeat significantly enhanced the cortisol and norepinephrine concentrations in blood. Enlargement of the spleen was also found in the social defeat and “social threat” groups. At the immunological level (Experiment 4), the social defeat group showed lower levels of pro-inflammatory cytokines in the hypothalamus and hippocampus but higher concentration of IL-6 in the striatum compared to the other two groups. At the neurochemical level (Experiment 5), the socially defeated hamsters mainly displayed reductions of dopamine, dopamine metabolites, and 5-HT levels in the striatum and decreased level of 5-HT in the hippocampus. In Experiment 6, an increase in the spine density of hippocampal CA1 pyramidal neurons was specifically observed in the “social threat” group. Collectively, our findings indicate that repeated, intermittent social defeats throughout entire adolescence in hamsters impact their adult responses at multiple levels. Our results also suggest that the “social threat” group may serve as an appropriate control. This study further suggest that the alterations of behavioral responses and neurobiological functions in the body and brain might provide potential markers to measure the negative consequences of chronic social defeats.

## Introduction

The juvenile period (or adolescence) is characterized by the rapid development of the brain and the vulnerability of continually maturing brain regions to stress, which influences behavioral, emotional, and cognitive functions in both humans and animals (Romeo and McEwen, 2006; McCormick and Mathews, 2010). Chronic stress during adolescence can lead to neuromorphological changes in specific brain regions, irregular inflammatory responses, and vulnerability to psychopathology in later life (Romeo and McEwen, 2006; Buwalda et al., 2011; Cohen et al., 2012). Among the daily-life stressors in adolescence, bullying (especially physical bullying) is a very common and severe stressor (Björkqvist, 2001; Bond et al., 2001; Klomek et al., 2007; Sourander et al., 2009). Intriguingly, social defeat can be considered an ecologically relevant animal model of physical bullying, and the effects of social defeat in animal models have increasingly shown parallels with those of bullying in humans (Björkqvist, 2001; Huhman, 2006).

There are an increasing number of studies on social defeat in animals, and the consequences of social defeat have been studied in multiple animal models. Exposure to repeated social defeat in adulthood not only enhanced anxiety-like behavior, depressive-like behavior, and hormonal responses (Kinsey et al., 2007; Yu et al., 2011; Shively and Willard, 2012) but also altered the monoaminergic system (especially dopamine and serotonin) and other aspects of the nervous system (Tidey and Miczek, 1996; Panksepp et al., 2007; Seo et al., 2008). Several studies further examined the behavioral and neurochemical consequences of experiencing repeated social defeat for a short period (e.g., 1–2 weeks) during adolescence on adult animals (Vidal et al., 2007; Watt et al., 2009; Burke et al., 2013). For example, the resident-intruder (or social disruption) paradigm has been established and used to segregate defeated mice into susceptible and unsusceptible populations, in which the resilience or coping strategy toward chronic social stress was reported to be regulated by the mesolimbic dopamine circuit (Krishnan et al., 2007). It was also reported that defeated mice in this behavioral paradigm increased the production of pro-inflammatory cytokines (e.g., IL-1β, IL-6, and TNF-α) in LPS-stimulated splenocyte cultures (Stark et al., 2001; Avitsur et al., 2002; Kinsey et al., 2007) and elicited alterations in the norepinephrine and serotonin levels within the prefrontal cortex (PFC), the hippocampus, and the amygdala (Jacobson-Pick et al., 2013). This behavioral paradigm, however, may not completely reflect a natural environment for social defeat (Tamashiro et al., 2005) because social defeats typically occur in a neutral social context or territory rather than in an animal's burrow.

In contrast to other laboratory animals used in social defeat models, the golden hamster (*Mesocricetus auratus*) is a classic model used to investigate the behavioral and neurobiological mechanisms underlying social interactions and social defeats for many years (Delville et al., 2003; Lai et al., 2005; Huhman, 2006; Solomon et al., 2007; Petrulis, 2009). The experience of social defeat has frequently been applied to hamsters to investigate the behavioral, physiological, hormonal, and neurobiological mechanisms of social conflict in adulthood (Wommack and Delville, 2003; Petrulis, 2009; Kuo et al., 2011), as well as the effects of such conflict on social learning and memory (Lai and Johnston, 2002; Lai et al., 2005, 2014; Johnston and Peng, 2008; Huang et al., 2011). Moreover, hamsters use cortisol as their primary glucocorticosteroid hormone (especially during the dark cycle or active phase) (Workman et al., 2011), and the stress hormones and responses of hamsters are considered to be similar to those of humans (Huhman et al., 1991). Importantly, unlike rats, puberty in both humans and hamsters is marked by a gradual activation of the Hypothalamic-Pituitary-Adrenal (HPA) axis, resulting in increasing baseline and stress-induced cortisol levels (Wommack et al., 2004, 2005). Thus, complementary to other existing animal models, the hamster model offers some features with respect to behavioral traits, physiological patterns, and several similar characteristics that facilitate the study of behavioral, hormonal, and neurobiological responses to experiencing social defeat.

In the present study, we utilized the agonistic behavior of male golden hamsters to develop a new behavioral model of repeated, sporadic social defeat across the entire juvenile period. Comparable to some human studies (e.g., Fekkes et al., 2005; Pepler et al., 2006), this model displays ecological validity in recapitulating some features of chronic physical bullying during human adolescence. A novel “social threat” group was also included in this study to evaluate the effect of “psychological” (non-physical) stress and the impact of indirect social exposure to aggressive adult hamsters. A set of six experiments was designed to comprehensively characterize the consequences of experiencing repeated, intermittent social defeats or “social threats” throughout the entire juvenile period on behavioral, physiological, hormonal, immunological, neurochemical and neuromorphological functions in young adult male hamsters. Integrating a top-down approach, we expected to observe changes consequential to repeated juvenile social defeats at multiple levels in young adult hamsters.

## Materials and methods

### General procedure and method

#### Animals

**Figure 1:**
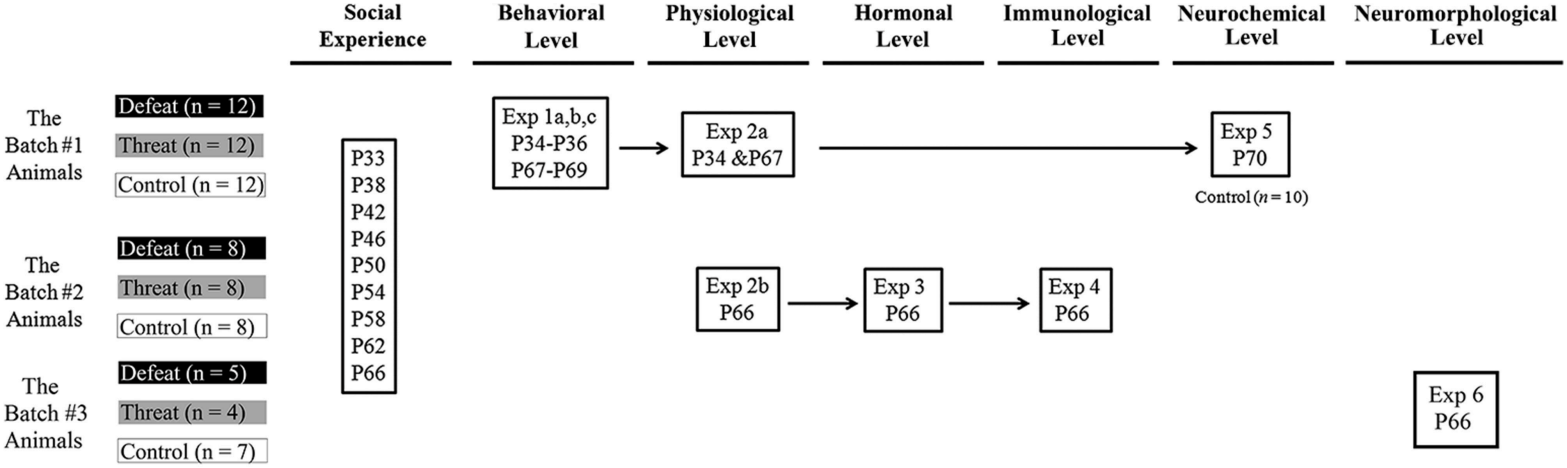
**The overall experimental design and animal use in this study**. A set of six experiments was conducted to investigate the consequences of repeated social defeats and “social threats” throughout the entire juvenile period on the behavioral, physiological, hormonal, immunological, neurochemical, and neuromorphological levels in young adulthood. Three batches of male juvenile hamsters were used in this study. There are three groups in each experiment: the social defeat group, the “social threat” group, and the arena control group. All males in each group received a series of nine social interaction trials (i.e., social defeat, “social threat”, or arena control conditions) in the social interaction chamber during the entire juvenile period, from postnatal day 33 to postnatal day 66. These time points were selected to correspond to the period from early puberty to young adulthood in hamsters and to recapitulate repeated, sporadic social defeats across the entire juvenile period in hamsters. P, postnatal day.

Juvenile male golden hamsters were used as subjects in this study. Additional adult males, 6–9 months of age, were trained with repeated winning experiences to serve as aggressive males and stimulus males in this study. As illustrated in Figure 1, a total of three batches of juvenile hamsters were used for a set of six experiments in this study. All of the hamsters were bred from the breeding stocks of the National Laboratory Animal Center in Taipei, Taiwan. They arrived at the animal facility of the Psychology Department, National Taiwan University, on postnatal day 21 and were temporarily group-housed before being housed individually in ventilated polysulfone cages (34 cm long × 22 cm wide × 16 cm high) containing corncob bedding a few days before the experiments. Food and water were provided *ad libitum*. The animal colony was maintained on a 12:12 h light:dark cycle with lights off at 8 AM and at a temperature of 22 ± 2°C. Animals were handled and weighed daily beginning 1 week before all experiments. All of the animal procedures were performed according to protocols approved by the appropriate Animal Care and Use Committees established by National Taiwan University. The minimum number of mice was used to meet the 3R reduction principle of animal use.

#### Apparatus

Three apparatuses were used in this study: a social interaction chamber, the home cage for the hamster, and a U-shaped maze. The social interaction chamber was a transparent acrylic chamber (20 × 22 × 19 cm) that was used to facilitate social interaction between two hamsters during the repeated social interaction trials for all 3 bathes of juvenile hamsters in all six experiments. The social interaction chamber was also used to assess the hamsters' spontaneous locomotor activity in two different non-social testing contexts in Experiment 1b. A transparent plastic lid was placed on the top of the chamber to prevent the hamsters from escaping. Each subject's home cage (34 × 22 × 16 cm) and a white metal mesh basket (8.5 × 8.5 × 14 cm) were used as a familiar social context to examine social investigation behaviors in Experiment 1a. The mesh basket was used to restrain a stimulus male in the home cage. Each subject's home cage was divided into three equally sized zones along the long axis (11 × 22 × 16 cm each). The proximal zone contained the metal basket with a restrained social stimulus male. The intermediate zone was in the middle of the cage, and the distal zone was the furthest zone from the stimulus male. The location of each subject was defined by the presence of its nose in one of the three zones. The third apparatus was an acrylic U-shaped maze; the details of this apparatus have been described elsewhere (Huang et al., 2011; Lai et al., 2014). This apparatus was designed as an unfamiliar social context to record behavioral responses to different stimulus males in Experiment 1c. All apparatuses, except the home cages of the animals, were cleaned with 70% alcohol before each trial.

#### Experimental manipulation and experimental design

All behavioral experiments were performed at least 1 h after the beginning of the dark cycle, and behavioral testing occurred under dim illumination (48 lux) in behavioral testing rooms that were separate from the animal colony. There were three groups in this study: the social defeat group, the “social threat” group, and the arena control group. Male hamsters at postnatal day 28 (P28) were randomly assigned to one of the three groups, and they correspondingly received a series of 9 social interaction trials (i.e., either social defeat, “social threat”, or arena control conditions) in the social interaction chamber on P33, P38, P42, P46, P50, P54, P58, P62, and P66. These time points were selected to correspond to the interval from early puberty to young adulthood in hamsters (Vomachka and Greenwald, 1979) and to recapitulate repeated, sporadic social defeats across the entire juvenile period (Pepler et al., 2006). Males in the social defeat and “social threat” groups received direct and indirect social encounters, respectively. For the social defeat group, a novel, aggressive adult male was placed in the social interaction chamber before introducing the subject during each social defeat trial. Each subject was allowed to have direct physical contact with the aggressive adult for 10 min. Dominance was rapidly established, and the adult male typically bit and chased the subject, who would try to escape from or jump out of the chamber. To control the duration of actual physical attack and to minimize the risk of severe injury in juvenile hamsters, each subject was held in a mesh metal basket (8.5 × 8.5 × 14 cm) after being cumulatively attacked (e.g., bitten in any part of the body or directly physically contacted/beaten up) for 30 s. The basket was removed 1 min before the end of the trial to promote physical contact again. The “social threat” group served as an indirect social encounter group that was allowed to indirectly interact with the stimulus males and explore the testing context. During each trial, males in the “social threat” group interacted with a novel, aggressive adult male that was restrained in the mesh metal basket within the social interaction chamber for 10 min for a series of nine sessions. The metal basket was used to avoid direct physical contact but facilitate indirect social interaction between the two males. Each social interaction trial used a different adult hamster with winning experience as the stimulus male to minimize habituation to the stimulus male. Males in the arena control group served as controls: each male was placed in the social interaction chamber alone for 10 min during the nine social interaction trials. Immediately after each social interaction trial, the approximate physical condition of each animal was assessed. No signs of significant fur loss or obvious bite wounds were found.

There were six experiments in this study. The experimental design and animal usage are illustrated in Figure 1. A total of three batches of juvenile hamsters were used. The hamsters in batch #1 were sequentially tested on behavioral consequences (Experiment 1), physiological changes (Experiment 2a), and neurotransmitter alterations (Experiment 5). The hamsters in batch #2 were tested on spleen index (Experiment 2b), peripheral hormonal alterations (Experiment 3) and pro-inflammatory cytokines in the brain (Experiment 4) after the last social interaction trial. The hamsters in batch #3 were tested on spine density of pyramidal neurons in hippocampus (Experiment 6) after the last social interaction trial. The details of each experiment are described below.

### Experiment 1 (behavioral level—batch #1 on P34-36 and P67-69): Evaluation of behavioral performance before and after the 1st (P33) and the 9th (P66) social interactions during the juvenile period in three different testing contexts

Three experiments (Experiment 1a–1c) were designed and conducted on hamster batch #1 consecutively. Males in each of the three groups (*n* = 12 each) received one of the three social interactions across the nine trials as described above. All males were separately and consistently habituated to each of the three testing apparatuses and the empty metal basket for 10 min each. All behaviors were recorded using ETHOM software (Shih and Mok, 2000) by an observer who was blinded to the subject's experimental group.

#### Experiment 1a—evaluation of behavioral performance in a familiar social context

Experiment 1a was designed to evaluate behavioral changes after experiencing the 1st and the 9th social interactions in a familiar context (i.e., the hamsters' home cages), where they were forced to defend their territories against a novel intruder (i.e., an aggressive adult hamster). Males in each of the three groups were tested on P29 and P62 for pre-test baselines before the 1st and 9th social interactions, respectively. They were tested again on P34 and P67 as post-tests after the 1st and 9th social interactions, respectively. Each behavioral test was composed of a 3-min no-stimulus trial and a 3-min stimulus trial in each subject's home cage. In the no-stimulus trial, a clean metal basket was placed at one end of the subject's home cage (defined as the proximal zone) for 3 min. Immediately after the end of the no-stimulus trial, a novel, experienced male intruder was confined in the metal basket, which was replaced at the same location in the subject's home cage for a 3-min stimulus trial. The time spent in each zone was recorded, and the total durations of three categories of behaviors were videotaped and recorded as described previously (Huang et al., 2011): (1) non-social behaviors consisting of exploration and grooming; (2) social sniffing behavior; and (3) submissive behaviors consisting of fleeing, head flagging, and avoidance.

#### Experiment 1b—evaluation of spontaneous locomotor activity in the familiar and unfamiliar non-social contexts

To evaluate behavioral responses in two different contexts where social interaction did or did not previously take place, this experiment was designed to further investigate the alterations of spontaneous locomotor activity in the familiar and unfamiliar non-social contexts after experiencing different social interactions in the social interaction chamber. Males in each of the three groups were tested on P30 and P63 (i.e., 1 day after behavioral test in Experiment 1a) for pre-test baselines before the 1st and 9th social interactions, respectively. They were tested again on P35 and P68 as post-tests after the 1st and 9th social interactions, respectively. Spontaneous locomotor activity was measured in the social interaction chamber using two different bedding materials (with different smells and textures) as distinct contextual cues. Each male was sequentially tested in an original (i.e., familiar) interacting context for a 3-min trial and in an altered (i.e., unfamiliar) testing context for another 3-min trial in a counterbalanced order. The original interacting context was the same as the context in which they underwent the social interaction trials previously. A different bedding material was inserted into the same chamber to create an altered testing context. Spontaneous locomotor activity was recorded and analyzed using the TopScan video tracking system (Clever Sys, Inc., VA, U.S.A.).

#### Experiment 1c—evaluation of behavioral changes in an unfamiliar social context

To further investigate the behavioral consequences of various social interactions in a novel, highly controlled social context, each subject was tested in a U-shaped maze before and after experiencing a given social interaction. Males in each of the 3 groups were tested on P31 and P64 (i.e., 1 day after the behavioral test in Experiment 1b) for pre-test baselines before the 1st and 9th social interactions, respectively. They were tested again on P36 and P69 for post-tests after the 1st and 9th social interactions, respectively. As adopted from previous studies (Huang et al., 2011; Lai et al., 2014), the behavioral test consisted of a 3-min no-stimulus trial and a 3-min stimulus trial. The 3-min stimulus trial began 1 min after the end of the no-stimulus trial. One predetermined stimulus animal (i.e., a novel hamster with experience in winning) was confined to the arm in which the subject had spent more time during the no-stimulus trial. During each trial, the subject was allowed to explore the U-maze for 3 min, and the time spent in each part of the U-maze was recorded using ETHOM software. Social investigation behavior was defined as the time spent in the basal part of the stimulus arm plus the screened area of the stimulus chamber as described previously (Lai et al., 2005, 2014).

### Experiment 2 (physiological level): Measurement of basic physiological changes after experiencing the 1st (P33) and the 9th (P66) social interactions during the juvenile period

#### Experiment 2a—measurement of fecal pellets and body weights (batch #1 on P34 and P67)

Given that fecal pellets and body weight can be used as non-invasive indexes for the measurement of basic physiological responses (Foster et al., 2006; Kuo et al., 2011), the number of fecal pellets and the body weight for all males in the three groups (*n* = 12 each from batch #1) were measured. To measure autonomic responses, the total numbers of fecal pellets voided by each subject were collected from the home cage of each subject for a total of 24 h before and after the 1st and the last social interaction trials. The procedure has previously been described in detail (Kuo et al., 2011). In addition, each subject's body weight was recorded 24 h after the first and the last social interaction trials to examine basic physiological development after experiencing different social interactions. The subjects were weighed during the dark cycle to prevent interference with each subject's daily rest and activity patterns.

#### Experiment 2b—measurement of the spleen index after experiencing nine social interaction trials (batch #2 on P66)

Because the spleen is an important organ in the immune system, the spleen weight was used as an index of physiological responses after experiencing 9 sessions of social interactions. The males of batch #2 from each of the three groups (*n* = 8 each from batch #2) were euthanized 30 min after the last social interaction trial, and their spleens were immediately harvested and weighed to prevent confounding due to water or body fluid loss. To normalize the differences in their body weights, the spleen index is expressed as spleen mass (mg)/body weight (g).

### Experiment 3 (hormone levels—batch #2 on P66): Examination of stress-related hormones in serum after experiencing nine social interaction trials

The subject's cortisol and sympathetic hormone levels were used as indexes of stress-related hormonal responses after experiencing nine sessions of social interactions. The batch #2 subjects (*n* = 8 per group) were used in this experiment. Blood samples were collected from the trunk of the animals and centrifuged at 3000 rpm at 4°C for 20 min. Sera were divided from blood and then stored at –80°C until use. The cortisol assay was performed using a cortisol ELISA kit (Enzo Life Sciences, Farmingdale, NY, U.S.A.), and the experimental procedure was modified from Wommack and Delville (2003). The ELISA plate was measured at 405 nm using a Multiskan FC microplate photometer (Thermo Fisher Scientific, Waltham, MA, U.S.A.), and the intra-assay variability was 15.6%. The cortisol level is presented as a concentration (ng/ml). The serum adrenaline and noradrenaline levels were measured using the 2-CAT (A-N) Research ELISA Kit (Labor Diagnostika Nord, Nordhorn, Germany). The adrenaline and noradrenaline ELISA plates were measured at 450 nm and a reference wavelength of 620 nm using a Multiskan FC. The intra-assay variability was 8.5% for adrenaline and 13.6% for noradrenaline. The levels of sympathetic hormones are presented as concentrations (ng/ml).

### Experiment 4 (immunological level—batch #2 on P66): Examination of the pro-inflammatory cytokine levels in different brain areas after experiencing nine social interaction trials

Because the expression levels of pro-inflammatory cytokines play a vital role in the activation of immune cells to promote inflammatory states, the expression levels of the cytokines TNF-α, IL-1β, and IL-6 were measured to reveal immune responses after experiencing nine sessions of social interactions. As described above, the males in batch #2 from each of the three groups (*n* = 8 each) were euthanized 30 min after the last social interaction trial. Tissue samples from several brain areas (including the PFC, the striatum, the thalamus, the hypothalamus and the hippocampus) were rapidly dissected and stored at –80°C until use. The cytokine levels in each brain area were assessed using the mouse Enhanced Sensitivity Flex Set Cytometric Bead Array and the BD Cytometric Bead Array Mouse Enhanced Sensitivity Master Buffer Kit (BD, San Diego, CA, U.S.A.). Brain tissues were extracted in lysis buffer consisting of 10 mM Tris in pH 8.0, 150 mM NaCl, 1 mM EDTA and 1% Triton X-100; this solution was modified from Chapman et al. (2009). Each tissue sample was treated with 300 μl of extraction buffer and 4 μl of protease inhibitor cocktail (Sigma, St. Louis, MO, U.S.A.). The supernatant was collected, followed by centrifugation for 15 min at 4°C at 12500 rpm. To prevent the lysis buffer from affecting the total assay reaction, a 3-fold dilution of the tissue sample was assayed in the presence of assay buffer, and 50 μl of standards and samples were inserted into the flow tube. After adding the reagents and washing step-by-step according to the instruction manual provided with the assay kit, samples were analyzed using a BD FACSCanto II flow cytometer (BD, San Diego, CA, U.S.A.). The cytokine levels were presented as standardized concentrations (fg cytokine/mg tissue).

### Experiment 5 (neurochemical level—batch #1 on P70): Evaluation of monoamine neurotransmitter levels in different brain areas after experiencing nine instances of social interaction

To characterize the effect of social interactions on the alterations of the levels of monoamine neurotransmitters and their metabolites in the brain, neurochemical analysis was conducted using high-performance liquid chromatography (HPLC). Batch #1 subjects from each of the three groups (*n* = 12 each) were euthanized 1 day after the final day of behavioral testing. The following brain regions of interest were surveyed and measured, including the PFC, the cortex (except the PFC), the striatum, the thalamus, the hypothalamus, the hippocampus and the cerebellum. The samples were rapidly frozen in liquid nitrogen and stored at –80°C until use. The concentrations of norepinephrine (NE), dopamine (DA), 3,4-dihydroxyphenylacetic acid (DOPAC), homovanillic acid (HVA), serotonin (5-HT), and 5-hydroxyindoleacetic acid (5-HIAA) were measured via HPLC coupled with an electrochemical detector (ECD, LC-4C, BAS, West Lafayette, IN, U.S.A.) and an autosampler (CMA 200 refrigerated microsampler, Stockholm, Sweden). The procedure has previously been described in detail (Hao et al., 2013). The monoamine concentrations for each sample were calculated according to the respective peak area using Peak-ABC software (Great Tide Instrument Co., Taipei, Taiwan). The neurotransmitter and metabolite levels are presented as concentrations (ng amine/g tissue).

### Experiment 6 (neuromorphological level—bath #3 on P66): Evaluation of spine densities in the hippocampal CA1 region after experiencing nine social interaction trials

Because previous studies have reported morphological changes to dendrites after the experience of stressors (Romeo and McEwen, 2006) and also because of our findings in Experiment 5, we expected to detect neuromorphological changes to dendritic spines of hippocampal neurons in response to long-term social defeats during the juvenile period. Batch #3 hamsters were euthanized immediately after the 9th social interaction. The hamsters were transcardially perfused with 0.9% saline followed by with 4% PFA. After perfusion, their brain tissues were collected and incubated in Golgi Stain kit solutions A and B in accordance with the instruction manual provided with the FD Golgi Stain kit (FD Neuro Technologies, Baltimore, MD, U.S.A.). The brain sections (150 μm) with silver staining were prepared and observed under a microscope at 100X magnification. Several images of each region were captured at different depths. Then, the spine density was calculated using ImageJ software. For the ease of analysis and the quality of staining, only pyramidal neurons in the CA1 region of dorsal hippocampus (around AP –1.5 mm from the Bregma) displaying high staining quality were selected. For each subject in each group, there are at least 15 segments (from at least two randomly labeled pyramidal neurons in the target region) that were scored and averaged for the spine density of the secondary and tertiary dendrites.

### Statistics and data analyses

All data are presented as the means ± standard error of the mean (SEM). The data that met the assumptions for normality and homogeneity of variance based on Kolmogorov–Smirnov tests were analyzed using parametric tests. All of the data were normally distributed (normality test, data not shown). Statistical evaluations were performed using one-way ANOVA or the two-sample Student's *t*-test, as appropriate, using SPSS 19.0 (SPSS Inc., Chicago, IL, U.S.A.). *Post-hoc* analysis was performed using Fisher's LSD test when the *F*-values revealed significant differences, and *p* < 0.05 were considered statistically significant.

## Results

### Experiment 1 (behavioral level—batch #1): evaluation of behavioral performance before and after the 1st and the 9th social interactions in three different testing contexts

#### Experiment 1a—behavioral changes in a familiar social context

**Figure 2:**
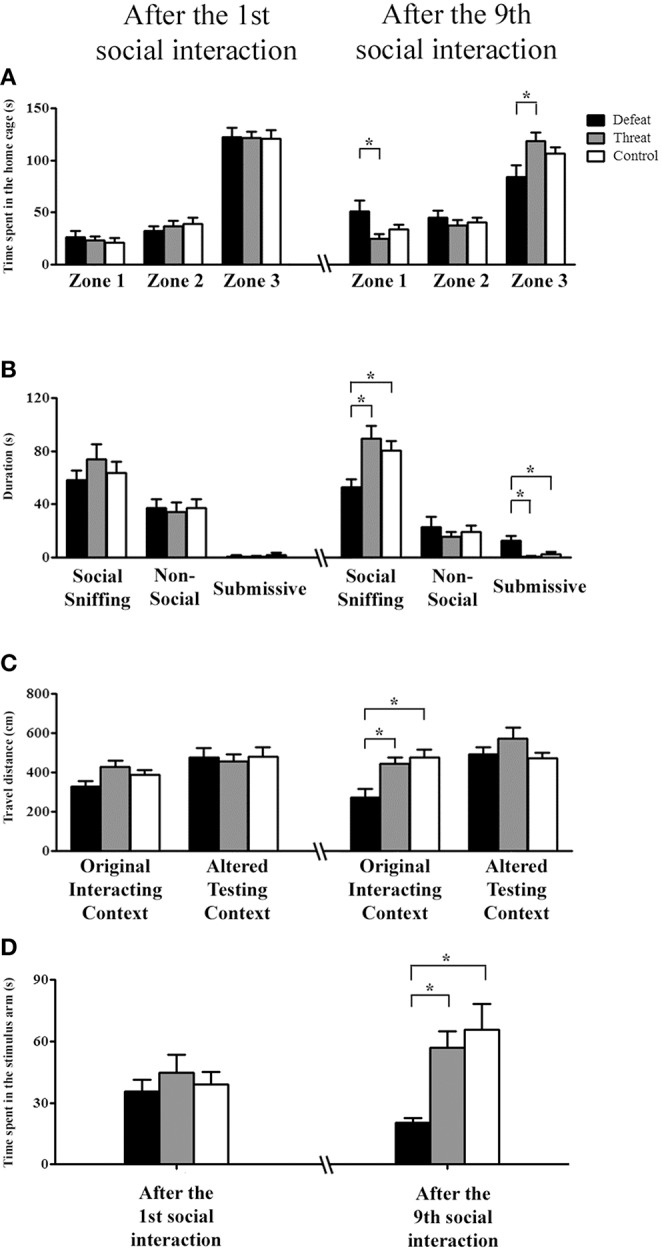
**The behavioral performances (means ± SEM) in the three different testing contexts of Experiment 1 were evaluated after experiencing the 1st (postnatal day 33, left panels) and the 9th (postnatal day 66, right panels) social interactions in the social defeat group (black bars), the “social threat” group (gray bars) and the arena control group (white bars, batch #1, *n* = 12 per group)**. **(A,B)** In Experiment 1a, the total time spent in each of the three zones and the total duration (s) of the three categorized behaviors in a familiar, social context (i.e., the subject's home cage) were recorded. Distal zone: the zone farthest from the stimulus male; intermediate zone: the zone in the middle of the home cage; and proximal zone: the zone containing the metal basket with a restrained social stimulus male. Behaviors were categorized as non-social behaviors, social sniffing behaviors, or submissive behaviors. **(C)** In Experiment 1b, spontaneous locomotor activity (cm) in a familiar, non-social context (i.e., the original interaction context) and in an unfamiliar, non-social context (i.e., the altered experimental context) was recorded. **(D)** In Experiment 1c, the total social investigation time (s) in the stimulus arm of the U-shaped maze (i.e., an unfamiliar social context) was recorded. ^*^p < 0.05.

Before the first social interaction, the three groups showed no significant differences in the pre-test results. After the first social interaction, neither the time spent in any of the three zones of the home cage (Figure 2A, left panel) nor the total duration of any of the three categories of behaviors (Figure 2B, left panel) differed significantly among the three groups. In contrast to the findings following the 1st social interaction, after nine social interaction sessions, the males in the social defeat group spent significantly more time in the distal zone [*F*_(2, 33)_ = 3.906, *p* = 0.03] and less time in the proximal zone [*F*_(2, 33)_ = 3.753, *p* = 0.034] than the males in the “social threat” group (Fisher's LSD: both *p* < 0.05; Figure 2A, right panel). Moreover, as depicted in Figure 2B (right panel), there were significant differences in the total duration of social sniffing behavior [*F*_(2, 33)_ = 6.198, *p* = 0.005] and submissive behavior [*F*_(2, 33)_ = 6.682, *p* = 0.004], but not that of non-social behavior [*F*_(2, 33)_ = 0.452, *p* = 0.640], among the three groups. Specifically, *post-hoc* analysis further revealed that the males in the social defeat group spent significantly less time performing social sniffing behavior and more time performing submissive behavior than the other two groups (all *p* < 0.05). Thus, after experiencing nine sessions of social defeats across the entire juvenile period, the hamsters in the social defeat group behaved differently toward an intruder in their home cage. They avoided spending time near the stimulus males and displayed more submissive behaviors only after experiencing repeated social defeats.

#### Experiment 1b—spontaneous locomotor activity in the original interacting and altered testing contexts

Before the first social interaction, the three groups showed no significant differences in the pre-test results. As shown in Figure 2C, the three groups also showed no significant differences in locomotor activity in either of the experimental contexts after the 1st social interaction. In contrast, after the 9th social interaction, one-way ANOVA revealed a significant difference in the original interacting context [*F*_(2, 33)_ = 7.24, *p* = 0.002], but not in the altered testing context [*F*_(2, 33)_ = 1.580, *p* = 0.221], among the three groups. *Post-hoc* comparisons further revealed that the males in the social defeat group exhibited significantly less locomotor activity in the original interacting context than did the other two groups (both *p* < 0.05; Figure 2C, right panel). However, no difference was observed between the “social threat” and arena control groups. These results indicate that the experience of repeated social defeats is sufficient to alter hamsters' spontaneous locomotion in their original interacting context, which was associated with repeated experiences of social defeat.

#### Experiment 1c—evaluation of the behavioral responses in an unfamiliar social context

On the U-shape maze test, no significant difference was found among the three groups before or after the 1st social interaction. After the 9th social interaction, the three groups showed a significant difference in the time spent in the stimulus arm [*F*_(2, 33)_ = 7.218, *p* = 0.003; Figure 2D]. *Post-hoc* comparisons further revealed that the males in the social defeat group spent less time investigating the stimulus male than did the males in the other two groups (both *p* < 0.05). However, no significant difference was observed in stimulus male investigation between the threat and control groups. These results indicate that male hamsters experiencing repeated social defeats exhibit decreased social investigation in an unfamiliar social context. Collectively, the results from Experiments 1a–c revealed that the experience of repeated social defeats (but not “social threats”) across the entire juvenile period significantly impacted locomotor activity in the familiar context and social interaction in both the familiar and unfamiliar social contexts.

### Experiment 2 (physiological level—2a: Bath #1; 2b: Bath #2): Measurement of physiological indexes

**Figure 3:**
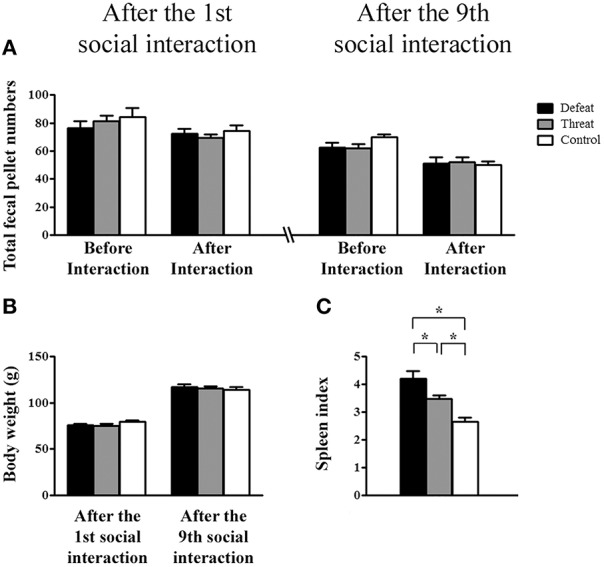
**Measurement of the basic physiological changes (means ± SEM) in the three groups (the social defeat group (black bars), the “social threat” group (gray bars), and the arena control group (white bars) after experiencing the 1st (left panels) and the 9th (right panels) social interaction trials across the entire juvenile period in Experiment 2**. **(A)** Total numbers of fecal pellets before and after the indicated social interaction trial in Experiment 2a (batch #1, *n* = 12 per group). **(B)** Body weight (g) after experiencing the 1st and 9th social interaction trials in Experiment 2a. **(C)** The spleen index [i.e., spleen mass (mg)/body weight (g)] after experiencing nine social interaction trials in Experiment 2b (batch #2, *n* = 8 per group). ^*^p < 0.05.

For the basic physiological indexes evaluated in Experiment 2a, as depicted in Figures 3A,B, neither the total number of fecal pellets nor the body weight showed significant differences among the three groups before or after the first or the 9th social interaction. As shown in Figure 3C, the spleen index differed significantly among the three groups based on the results from Experiment 2b [*F*_(2, 21)_ = 15.736, *p* < 0.05]. *Post-hoc* comparisons revealed that the social defeat group had a significantly higher spleen index than the other two groups and that the “social threat” group had a significantly higher spleen index than the control group (all *p* < 0.05). Thus, both repeated social defeats and “social threats” resulted in the enlargement of the spleen index in young adults, especially in the social defeat group.

### Experiment 3 (hormone levels—batch #2): Examination of stress-related hormones in serum after experiencing the 9th social interaction

**Figure 4:**
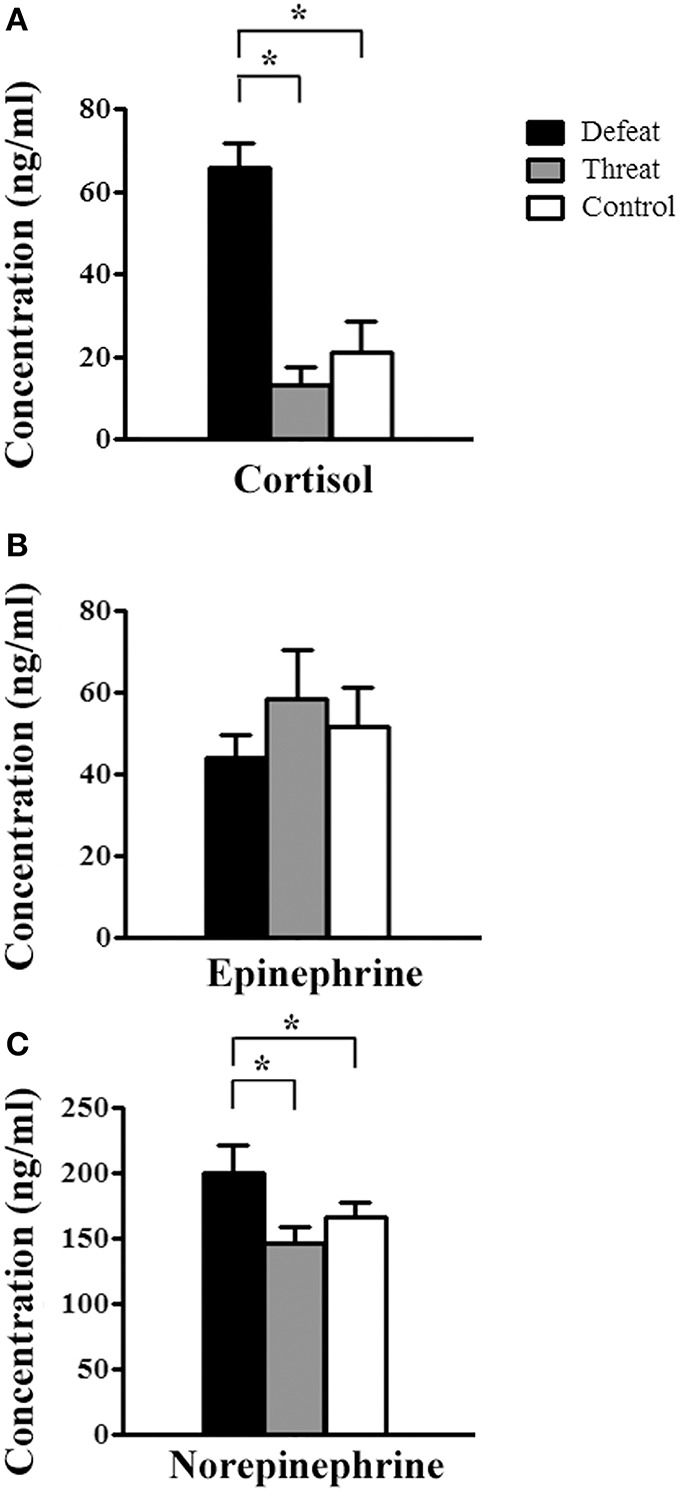
**The alterations in the serum levels of stress-related hormones (means ± SEM ng/ml) in the three groups (batch #2, *n* = 8 each) after experiencing nine social interaction trials in Experiment 3**. **(A)** Serum cortisol concentration. **(B)** Serum epinephrine concentration. **(C)** Serum norepinephrine concentration. ^*^p < 0.05.

As depicted in Figure 4, after the 9th social interaction, there were significant differences in the cortisol [*F*_(2, 21)_ = 23.037, *p* < 0.001] and norepinephrine concentrations [*F*_(2, 21)_ = 7.599, *p* = 0.003], but not in the epinephrine concentration [*F*_(2, 21)_ = 0.004, *p* = 0.996], among the three groups. *Post-hoc* comparisons further revealed that the young adult male hamsters in the social defeat group displayed significantly higher cortisol and norepinephrine concentrations than the other two groups (all *p* < 0.05, Figures 4A,C, respectively).

### Experiment 4 (immunological level—batch #2): Examination of the pro-inflammatory cytokine levels in different brain areas after experiencing nine social interaction trials

The concentrations of the three pro-inflammatory cytokines are shown in Table 1. Among the cytokines that we examined, significant between-group differences were found in the striatum, the hypothalamus and the hippocampus but not in the PFC or the thalamus. In the striatum, a significant between-group difference was found in the expression of IL-6 [*F*_(2, 21)_ = 4.166, *p* = 0.03] but not TNF-α [*F*_(2, 21)_ = 2.56, *p* = 0.101] or IL-1β [*F*_(2, 21)_ = 0.523, *p* = 0.6]. *Post-hoc* comparisons revealed that the social defeat group displayed a higher concentration of IL-6 than the control group (*p* < 0.05). In the hypothalamus, significant between-group differences were found in the expression of IL-6 [*F*_(2, 21)_ = 6.762, *p* = 0.005] and IL-1β [*F*_(2, 21)_ = 9.466, *p* = 0.001] but not TNF-α [*F*_(2, 21)_ = 0.009, *p* = 0.991]. *Post-hoc* comparisons revealed that the social defeat group displayed significantly lower concentrations of both IL-6 and IL-1β than the other two groups (all *p* < 0.05). In the hippocampus, significant between-group differences were found in the expression of TNF-α [*F*_(2, 21)_ = 3.691, *p* = 0.042] and IL-1β [*F*_(2, 21)_ = 5.729, *p* = 0.01] but not IL-6 [*F*_(2, 21)_ = 2.254, *p* = 0.13]. *Post-hoc* comparisons revealed that the social defeat group displayed lower concentrations of TNF-α and IL-1β than the control group (*p* < 0.05). These results indicated that the experience of long-term social defeats exerted differential effects on the expression of these three pro-inflammatory cytokines in distinct brain areas.

**Table 1:** **Early adulthood (postnatal day 66) levels (Mean ± SEM fg/mg tissue) of cytokines in different brain areas following social defeat or “social threat” across the entire juvenile stage in Experiment 4 (batch #2, *n* = 8 per group)**.

**Brain areas**	**Social experience**	**TNF-α**	**IL-6**	**IL-1β**
Prefrontal cortex	Social defeat	5.3 ± 1.5	7.3 ± 2.6	15.6 ± 4.0
	“Social threat”	9.2 ± 3.1	9.8 ± 1.8	6.5 ± 2.9
	Control	8.5 ± 4.9	13.4 ± 5.0	19.0 ± 5.3
Striatum	Social defeat	57.3 ± 12.4	100.999 ± 22.5^*^	17.8 ± 9.9
	“Social threat”	32.6 ± 12.8	60.292 ± 7.6	7.8 ± 4.0
	Control	23.0 ± 6.9	46.405 ± 4.2	16.7 ± 7.6
Thalamus	Social defeat	10.9 ± 2.7	30.5 ± 2.3	84.5 ± 12.0
	“Social threat”	12.2 ± 7.6	24.8 ± 7.5	74.4 ± 15.6
	Control	18.8 ± 4.9	37.3 ± 6.2	89.1 ± 19.6
Hypothalamus	Social defeat	11.5 ± 4.0	20.3 ± 3.9^*^^#^	52.3 ± 11.6^*^^#^
	“Social threat”	12.3 ± 5.6	46.9 ± 5.9	115.7 ± 23.8
	Control	12.0 ± 3.2	42.5 ± 6.3	162.0 ± 16.2
Hippocampus	Social defeat	0^*^	7.2 ± 1.8	1.8 ± 1.2^*^
	“Social threat”	2.2 ± 1.7	8.7 ± 2.4	8.2 ± 2.8
	Control	6.2 ± 2.2	18.5 ± 6.4	14.2 ± 3.1

* *Significant difference between social defeat and control group (p < 0.05)*.

# *Significant difference between “social threat” and control group (p < 0.05)*.

### Experiment 5 (neurochemical level—batch #1): Evaluation of the levels of neurochemicals in different brain areas after experiencing nine social interaction trials

The concentrations of monoamines and their metabolites in different brain areas are presented in Table 2. Among all of the monoamines and monoamine metabolites that we examined, there were no significant between-group differences in the neurochemical levels in the PFC, the thalamus, the hypothalamus, the cerebellum, or the cortex among the three groups. In contrast, there were significant between-group differences in the neurochemical levels in the striatum and the hippocampus between the three groups. In the striatum, significant between-group differences in the concentrations of dopamine [*F*_(2, 31)_ = 3.759, *p* = 0.035], DOPAC [*F*_(2, 31)_ = 4.14, *p* = 0.025], HVA [*F*_(2, 31)_ = 5.180, *p* = 0.011], 5-HT [*F*_(2, 31)_ = 3.606, *p* = 0.039], and 5-HIAA [*F*_(2, 31)_ = 7.751, *p* = 0.002] were observed. *Post-hoc* comparisons further revealed that the males in the social defeat group displayed significantly lower concentrations of dopamine, DOPAC, HVA, and 5-HIAA than those in the other two groups (all *p* < 0.05) and displayed a significantly lower concentration of 5-HT than those in the “social threat” group (*p* < 0.05). However, no significant difference was found in the turnover rates of DA, 5-HT, and NE among the three groups. In the hippocampus, the only significant difference in neurochemical levels was in the concentration of 5-HT [*F*_(2, 31)_ = 4.837, *p* = 0.015]. A marginally significant difference in the concentration of 5-HIAA [*F*_(2, 31)_ = 2.987, *p* = 0.06] was detected among the three groups. *Post-hoc* comparisons further revealed that the males in the social defeat group displayed a significantly lower concentration of 5-HT than the other two groups (both *p* < 0.05). These results indicate that the experience of repeated social defeat exerted a region-specific impact on the neurotransmitter levels in the brains of these young adult hamsters, especially in the striatum and the hippocampus.

**Table 2:** **Summary of levels (Mean ± SEM ng/g tissue) of monoamines (Norepinephrine, Dopamine and Serotonin [5-HT]), and metabolites (3,4-Dihydroxyphenylacetic acid [DOPAC], Homovanillic acid [HVA], and 5-Hydroxyindoleacetic acid [5-HIAA]) in different brain areas after experiencing chronic social defeat or “social threat” in juvenile stage (Experiment 5, batch #1, social defeat group: *n* = 12; ”social threat” group: *n* = 12; arena control group: *n* = 10)**.

**Brain areas**	**Social experience**	**Norepinephrine**	**Dopamine**	**DOPAC**	**HVA**	**DA turnover**	**5-HT**	**5-HIAA**	**5-HT turnover**
Prefrontal cortex	Social defeat	323.65 ± 23.16	37.45 ± 6.45	11.15 ± 2.07	10.60 ± 3.94	0.58 ± 0.28	348.26 ± 71.70	447.49 ± 32.05	1.29 ± 0.14
	“Social threat”	343.17 ± 7.92	42.75 ± 5.79	8.94 ± 0.84	8.03 ± 1.41	0.40 ± 0.11	326.34 ± 31.64	450.21 ± 43.17	1.38 ± 0.39
	Control	391.93 ± 11.25	45.65 ± 5.47	11.10 ± 1.66	13.39 ± 3.40	0.54 ± 0.29	339.83 ± 17.33	463.01 ± 28.85	1.36 ± 0.53
Striatum	Social defeat	490.43 ± 108.42	6782.57 ± 2233.74^*^^#^	786.57 ± 119.49^*^^#^	445.59 ± 83.25^*^^#^	0.18 ± 0.03	749.42 ± 133.81^#^	486.22 ± 66.08^*^^#^	0.65 ± 0.14
	“Social threat”	654.95 ± 132.05	16789.84 ± 2832.24	1244.99 ± 125.29	815.71 ± 90.67	0.12 ± 0.02	1289.36 ± 153.81	766.23 ± 62.64	0.59 ± 0.12
	Control	523.43 ± 109.36	15798.59 ± 3694.61	1315.47 ± 186.70	809.34 ± 112.25	0.13 ± 0.03	1158.39 ± 170.56	808.29 ± 59.10	0.70 ± 0.11
Thalamus	Social defeat	2122.27 ± 332.51	16317.99 ± 2373.14	1002.24 ± 131.39	814.60 ± 87.25	0.11 ± 0.03	1916.50 ± 276.72	1035.16 ± 103.13	0.54 ± 0.11
	“Social threat”	1542.74 ± 256.08	11664.96 ± 1241.12	704.01 ± 122.31	611.21 ± 66.07	0.11 ± 0.04	1624.22 ± 142.48	910.88 ± 74.73	0.56 ± 0.15
	Control	1832.22 ± 167.46	10454.32 ± 1411.48	732.14 ± 108.86	577.40 ± 71.88	0.13 ± 0.04	1532.94 ± 126.57	988.58 ± 62.35	0.65 ± 0.16
Hypothalamus	Social defeat	5458.33 ± 541.40	3563.34 ± 818.89	352.92 ± 65.58	323.15 ± 56.38	0.19 ± 0.04	2241.04 ± 209.53	1353.11 ± 114.77	0.60 ± 0.16
	“Social threat”	5646.85 ± 704.19	4450.20 ± 1476.81	435.86 ± 97.86	379.33 ± 84.87	0.18 ± 0.04	2571.49 ± 237.12	1571.01 ± 134.93	0.61 ± 0.17
	Control	6324.84 ± 811.75	2228.77 ± 938.38	265.90 ± 78.05	308.91 ± 91.64	0.26 ± 0.06	2415.33 ± 231.68	1598.98 ± 149.96	0.66 ± 0.21
Hippocampus	Social defeat	1056.96 ± 35.39	27.76 ± 2.87	14.74 ± 1.05	8.32 ± 1.57	0.83 ± 0.26	1321.74 ± 47.21^*^^#^	663.75 ± 27.55	0.50 ± 0.17
	“Social threat”	1094.39 ± 36.39	21.59 ± 1.95	12.29 ± 0.66	12.39 ± 2.39	1.14 ± 0.45	1452.13 ± 32.12	718.48 ± 20.28	0.50 ± 0.18
	Control	1080.59 ± 33.77	23.63 ± 3.61	12.90 ± 0.72	6.66 ± 1.26	0.83 ± 0.17	1475.86 ± 28.94	751.36 ± 28.22	0.51 ± 0.31
Cerebellum	Social defeat	185.97 ± 15.76	144.39 ± 18.37	3.44 ± 0.17	3.24 ± 0.70	0.05 ± 0.01	78.25 ± 4.00	88.97 ± 3.85	1.14 ± 0.28
	“Social threat”	189.80 ± 16.10	121.98 ± 17.98	5.91 ± 2.76	2.95 ± 0.29	0.08 ± 0.01	85.26 ± 4.12	92.03 ± 2.17	1.09 ± 0.02
	Control	182.68 ± 16.29	97.94 ± 18.60	6.61 ± 3.60	2.91 ± 0.32	0.10 ± 0.07	73.05 ± 10.71	93.03 ± 5.25	1.27 ± 0.16
Cortex	Social defeat	N. D.	472.82 ± 139.62	19.18 ± 4.35	20.11 ± 5.71	0.08 ± 0.02	704.44 ± 74.79	251.46 ± 18.10	0.36 ± 0.07
	“Social threat”	N. D.	272.54 ± 78.85	14.72 ± 2.01	15.79 ± 4.42	0.11 ± 0.02	716.18 ± 75.57	281.43 ± 21.75	0.39 ± 0.08
	Control	N. D.	246.18 ± 69.23	12.98 ± 1.68	9.92 ± 2.87	0.09 ± 0.02	740.67 ± 96.77	298.06 ± 27.79	0.40 ± 0.09

* *Significant difference between social defeat and control group (p < 0.05)*.

# *Significant difference between “social threat” and control group (p < 0.05)*.

### Experiment 6 (neuromorphological level—bath #3): Evaluation of the neuromorphological changes in hippocampal CA1 pyramidal neurons after experiencing nine social interaction trials

**Figure 5:**
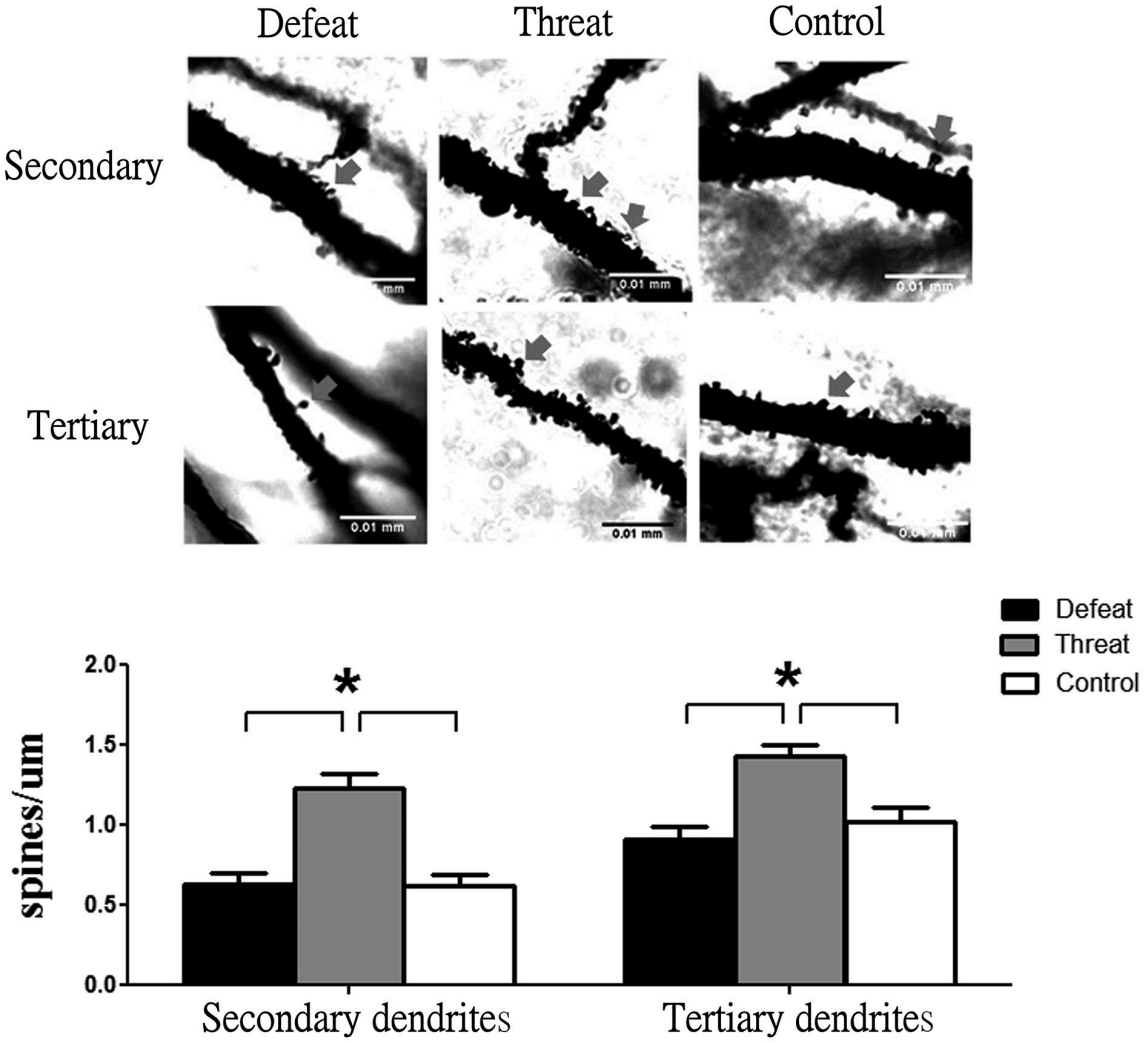
**Representative images and spine densities of CA1 pyramidal neurons in the three groups (batch #3) after experiencing nine social interaction trials across the entire juvenile period in Experiment 6**. A total of 15 segments in the target region were scored and averaged for each group. Scale bar = 0.01 mm; ^*^p < 0.05.

Given that the hippocampus is a brain region that continuously develops throughout adolescence and is closely associated with learning and memory (Romeo and McEwen, 2006) and given that we found neurochemical alterations in the hippocampus in Experiment 5, we further investigated the spine density of pyramidal neurons in the CA1 region of dorsal hippocampus. As shown in Figure 5, after experiencing nine social interaction trials, the hamsters in the “social threat” group exhibited higher densities of dendritic spines in both secondary and tertiary dendrites than hamsters in the social defeat and control groups [*F*_(2, 42)_ = 20.672, *p* < 0.05]. However, no significant difference in dendritic spine density was observed between the social defeat and control groups.

## Discussion

Our main findings can be summarized as follows. First, at the behavioral level (Experiment 1), the experience of repeated, sporadic social defeats (but not “social threats”) across the entire juvenile period significantly impacted locomotor activity in the familiar context and social interaction in both the familiar and unfamiliar social contexts. Second, at the physiological and hormonal levels (Experiments 2 and 3), repeated social defeat significantly enhanced the cortisol and norepinephrine concentrations in blood and the spleen index. Third, at the immunological level (Experiment 4), the social defeat group showed lower levels of pro-inflammatory cytokines in the hypothalamus and hippocampus but higher concentration of IL-6 in the striatum compared to the other two groups. Forth, at the neurochemical level (Experiment 5), the socially defeated hamsters mainly displayed reductions of dopamine, dopamine metabolites, and 5-HT levels in the striatum and decreased level of 5-HT in the hippocampus. Last but not least, at the neuromorphological level (Experiment 6), an increase in the spine density of hippocampal CA1 pyramidal neurons was specifically observed in the “social threat” group. In brief, in this study, we found that the defeated hamsters showed significant differences from the arena controls at multiple levels (Experiments 1–5), and the “social threat” group showed significant differences from the controls in the spleen index and the hippocampal spine densities (Experiments 2 and 6).

The golden hamster is well-accepted as a model for studying social defeat, conditioned defeat, and social learning and memory (Lai and Johnston, 2002; Delville et al., 2003; Lai et al., 2005, 2014; Huhman, 2006; Solomon et al., 2007; Petrulis, 2009). In this study, a procedure of repeated, intermittent social defeats and “social threat” across the entire juvenile period (i.e., from P33 to P66) was developed and applied to evaluate its long-term effects in young adulthood and to recapitulate some features of chronic physical bullying during human adolescence. At the behavioral level (Experiment 1a–1c), the behavior of avoiding the investigation of other hamsters, even in their own territory (i.e., their home cages), is similar to social withdrawal symptoms observed in human victims of bullying. It has been reported that hamsters showed anxiety-like behaviors specific to unfamiliar adult hamsters but did not exhibit a generalized anxious state in the absence of a social stimulus after repeated social defeats from P28 to P42 (Bastida et al., 2009). This finding is consistent with our results from the social contexts of Experiment 1a and 1c. Our results further indicate that the socially defeated hamsters displayed a reduction of spontaneous locomotion only in the familiar context, where the defeat took place, but not in an unfamiliar context (Experiment 1b). They also showed decreased social investigation time toward an unfamiliar adult male in an unfamiliar social context (Experiment 1c). These findings suggest that environmental cues play a crucial role in social defeat and that the generalized anxious state is social-dependent and context-specific in these defeated young adult hamsters. Interestingly, in adult male hamsters (>4 months of age), a single social defeat resulted in differential social investigation time toward familiar and unfamiliar opponents and the defeated adult hamsters also displayed submissive behaviors when facing a familiar opponent in both familiar and unfamiliar contexts (Lai and Johnston, 2002; Huang et al., 2011). In contrast, adult hamsters that experienced repeated (i.e., 10 times) social defeats rather than a single social defeat displayed an anxiogenic effect in a novel, non-social context (Huang et al., 2011), which is different from our current finding in the socially defeated young adult hamsters. Our result suggests that juvenile and adult hamsters show distinct effects of repeated social defeat. For the single social defeat on P33, given the findings that play-fighting peaks between P30 and P35 in golden hamsters (Pellis and Pellis, 1988; Wommack and Delville, 2003), it is not surprising to find that a single social defeat had no significant effect on behavioral responses of hamsters during the early juvenile stage.

In addition to the behavioral changes in Experiment 1, physiological responses are well-known to be altered by stressful experiences via the sympathetic nervous system (SNS) and the HPA axis (Tamashiro et al., 2005; Denver, 2009). Stress-induced autonomic responses and hormonal changes have been reported to affect anxiety, physiological responses (e.g., defecation), and body weight (Tamashiro et al., 2005; Pervanidou and Chrousos, 2012). It was reported that repeated severe social defeats affect food intake and body mass in adult male hamsters (Foster et al., 2006) but not in juvenile hamsters (Wommack et al., 2004). Consistent with the previous result in juveniles, our hamsters, which underwent sporadic social defeats or “social threats” throughout the entire juvenile period, did not exhibit apparent changes in body weight or defecation based on the results from Experiment 2. The absence of pronounced weight gain in defeated juvenile hamsters could be due to the differences in experimental procedure or the rapid growth during the juvenile period. Moreover, our data regarding the levels of stress hormones (including cortisol and epinephrine levels) from Experiment 3 are also in line with the results of other related studies in adolescent male rats and hamsters (Wommack and Delville, 2003; Watt et al., 2009; Hanke et al., 2012). These results support the hypothesis that repeated social defeat exerts distinct impacts on the physiological responses of adolescent and adult hamsters. The function and secretion of metabolism-related hormones (e.g., growth hormone, thyroid hormone, ghrelin, neuropeptide Y, and insulin) at different developmental stages may explain these discrepancies between adolescent and adult hamsters.

Intriguingly, Experiment 2b showed significant alterations in the spleen index and enhancements were detected for both the social defeat group and the “social threat” group after repeated social interaction. The indirect exposure to a novel, aggressive adult male can be considered as a threatening stressor because the golden hamster is a territorial animal that consistently fights against conspecifics for territorial dominance (Lai and Johnston, 2002; Huhman, 2006). To prevent unwanted restrained stress and to facilitate behavioral observation and recording, subjects in this group were allowed to freely move around for indirect social encounter with an aggressor. In most of our experiment, our “social threat” group did not exhibit significant differences from the controls, suggesting that a further modification may be needed to reveal the impact of a real “social threat”. Regardless, it is evident that the repeated “social threats” resulted in an increased spleen index, confirming that the “social threat” group may serve as a more appropriate control group than the arena control group (i.e., no stimulus animal in the testing arena) *per se*. Intriguingly, an enhancement in dendritic spine density was only found in the “social threat” group (Experiment 6). It is possible that the experience of repeated “social threats” provided additional stimuli for social enrichment throughout the juvenile period, and such stimuli may facilitate the genesis of dendritic spines in CA1 pyramidal neurons, as reported previously in related studies focused on environmental enrichment (e.g., Faherty et al., 2003). However, excessive exposure to social stimuli or social defeats might result in an increase in the levels of stress hormones, which can lead to the suppression of dendrite spine formation. Further studies are needed to clarify this possibility.

Moreover, the finding in the spleen might be relevant to the observed abnormalities of the levels of brain pro-inflammatory cytokines in the results from Experiment 4. The alteration of cytokine levels in the hypothalamus is of interest because the hypothalamus is a brain region that directly interacts with peripheral cytokine signals (Miller et al., 2009). Previous studies revealed that HPA axis activity is associated with cytokine expression, both peripherally and centrally (Hayley et al., 1999; Anisman et al., 2008). It has also been reported that long-term stressful life caused a decrease in the sensitivity of immune cells to glucocorticoid hormones, thus interfered with the down-regulation of pro-inflammatory cytokines response (Cohen et al., 2012). In this study, a region-specific opposite pattern was found between certain pro-inflammatory cytokines in some brain areas and the stress hormone levels in the periphery. Our results showed that the socially defeated hamsters showed lower levels of cytokines in the hypothalamus and the hippocampus but higher levels of cortisol and norepinephrine in peripheral blood. Compared with the result reported by (Jasnow et al., 2001), one possible explanation for the opposite pattern between brain cytokine levels and the stress hormone levels in the periphery is that there are distinct region-specific immunological effects in response to different types of stressors. Moreover, the expression levels of IL-1β, IL-6, and TNF-α might affect behaviors by altering neurotransmitter functions in the brain. For example, it has been reported that subchronic IL-1β infusion can affect neurotransmitter expression, particularly increasing the accumulation of 5-HT metabolites in the hippocampus (Anisman et al., 2008). In the present study, reductions in the IL-1β and 5-HT concentrations in the hippocampus were found in hamsters that underwent repeated social defeats. It has been reported that pro-inflammatory cytokines can activate indoleamine–2,3–dioxygenase (IDO) (Pemberton et al., 1997; Fujigaki et al., 2006), which catabolizes tryptophan into kynurenine, to reduce the serotonin concentration and to affect dopamine, acetylcholine and NMDA function (Schwarcz and Pellicciari, 2002). Thus, it is possible that the alterations in the levels of monoamines in the hippocampus and the striatum of the socially defeated hamsters resulted from changes in the levels of cytokines after experiencing repeated social defeats. Further examination of IDO activity and the kynurenine/serotonin ratio after experiencing long-term social defeats is warranted in a future study.

In addition, significant reductions in the levels of monoamine neurotransmitters were found in the striatum and the hippocampus of the socially defeated hamsters (Experiment 5). Dopamine and serotonin have been implicated in the regulation of aggressive behavior (van Erp and Miczek, 2000; Seo et al., 2008). The striatum has been identified as a key area in which dopaminergic systems are involved in motor control and in reward-based learning (Joel and Weiner, 2000), and its maturation is considered to be related to behavioral transitions from adolescence to adulthood (Casey and Jones, 2010). The resilience or coping strategy toward chronic social stress in mice was also reported to be regulated by the mesolimbic dopamine circuit, especially in the ventral striatum (Krishnan et al., 2007). The reduction of the serotonin level has been correlated with depression and aggressive behavior in both humans and animals (Ferrari et al., 2006; Popova, 2006). In this study, the socially defeated hamsters exhibited reductions of dopamine, dopamine metabolites, and 5-HT levels in the striatum. It has been shown that repeated low-dose cocaine treatment during adolescence facilitates offensive aggression in male golden hamsters (DeLeon et al., 2002), supporting the involvement of dopamine in the regulation of aggression. Intriguingly, it was reported that BDNF (brain-derived neurotrophic factor) signaling within the mesolimbic dopamine circuit relates to vulnerability and resistance to social defeat in the resident-intruder paradigm in mice (Krishnan et al., 2007), which might be somewhat related to and support our current findings in the striatum. The precise role of BDNF and dopaminergic system in the striatum warrant further investigation using our repeated social defeat model in hamsters. Besides, 5-HT has also been implicated in the control of aggression in both humans and animal models (DeLeon et al., 2002; Seo et al., 2008), and 5-HT activity in the anterior hypothalamus has been shown to regulate/inhibit aggressive behavior in golden hamsters (Ferris et al., 1999). However, in our current social defeat (instead of aggression) model in young adult hamsters, significant reductions of 5-HT were found in the striatum and hippocampus, but not in the hypothalamus. This result suggests that 5-HT in different brain areas might serve distinct roles in the regulation of aggression and social defeat. Furthermore, a context-dependent alteration of locomotor activity was only found in the social defeat group of this study, suggesting that the reductions of neurotransmitters in the striatum and the hippocampus are related to social functions rather than to general motor functions. The prolonged decreases in the levels of neurotransmitters and their metabolites in these brain regions might be involved in the establishment of poor social functions and behavioral deficits in adulthood. The cooperative interactions between hippocampus and striatum on repeated social defeats warrants further investigation.

There are some limitations of this study. First, although animal models of social defeat appear to be advantageous for clarifying the underlying effects of social defeats, findings in any animal model are limited regarding their application to human bullying. And additional caution should be made when comparing animal data with bullying in humans. Second, our model concentrated on the effects of repeated, sporadic social defeats across the entire juvenile period instead of any specific developmental stage during adolescence, which may not be completely comparable to some existing data using similar but different behavioral models. Third, due to the limitations of our animal facility, our hamsters were housed in a sub-optimal photoperiod, which may somewhat delayed their sexual maturation. However, considering the total experimental period and the main purpose of this study, the long period of social defeats applied from P33-P66 might eliminate or minimize the effect of the plausible limitation. Compared to our previous data and other relevant studies, we did not notice any significantly behavioral difference or reduced body weight in our young adult hamsters. But it will be favorable to work out a reasonable method to measure their physical maturity and sexual maturation over the course of development in the future study. Fourth, the biochemical and morphological analyses were conducted in limited brain regions. For example, it has been reported that cortical areas, amygdala, and subregions of hippocampus are involved in social learning and conditioned defeat in hamsters (Lai et al., 2005; Huhman, 2006; Petrulis, 2009; Jacobson-Pick et al., 2013). Examining additional brain areas would be interesting in future studies. Nevertheless, our hamster model offers several advantages in terms of experimental validity and utility, and it provides an integrated approach to obtaining a comprehensive understanding of the consequences of long-term social defeats. Repeated stress-induced alterations in cytokine and monoamine levels could represent potential mechanisms to explain the behavioral alterations observed in early adulthood. The rebalancing of immune function and of the monoamine levels might provide markers to support the alleviation of the negative consequences of repeated social defeats.

Overall, our current repeated, intermittent social defeat model might complement many previous animal models and findings by providing additional evidence relevant to both basic and translational research. First, most animal models of social defeat have typically applied consecutive daily social defeats for a short period, and this schedule may not completely reflect the frequency of bullying events in some victims of bullying (e.g., once or several times per week for a long period of time; Fekkes et al., 2005). Second, bullying events among humans usually decrease from early to late adolescence but occur continuously in late adolescence (Pepler et al., 2006). Accordingly, a series of specific time points corresponding to the whole period from early puberty to young adulthood (Vomachka and Greenwald, 1979) was selected for repeated, sporadic social interactions in the current study. Third, the resident-intruder paradigm is a widely used animal model of social defeat (Björkqvist, 2001; Krishnan et al., 2007), but the defeated experience used typically occurs in the resident's home cage or territory, unlike the location of social defeat among wild animals and humans. Fourth, an integrated approach was used in this study to produce results at multiple levels, providing a comprehensive understanding of the effects. Last but not least, in addition to using home cage animals or arena control animals as a control group, a “social threat” group was included in this study to serve as a proper control for indirect social exposure. Thus, many follow-up experiments can be carried out in the near future based on these interesting findings in this study. Commentary to human studies, this model could be used to further characterize behavioral, hormonal, and neurobiological responses to experiencing repeated social defeats, which might shed light on our understanding of social defeat in humans and on the development of new treatments for victims of physical bullying.

## Author contributions

WY conducted experiments 1–5, analyzed data, and wrote up the first draft of this manuscript. CL conducted experiment 6, performed statistical analysis, prepared figures and tables, and revised MS. WL designed all experiments, supervised all experiments, modified MS, revised MS, and submitted MS.

### Conflict of interest statement

The authors declare that the research was conducted in the absence of any commercial or financial relationships that could be construed as a potential conflict of interest.
